# Innovative Digital Cognitive Behavioral Treatment for Insomnia Disorder in Adults (dCBT-i): Framework Development

**DOI:** 10.2196/70193

**Published:** 2025-05-27

**Authors:** Emilie Stern, Severine Brune, Stéphane Mouchabac, Anthony Dubroc, Charles de la Personne, Pierre Alexis Geoffroy

**Affiliations:** 1 Centre ChronoS GHU Paris psychiatrie & neurosciences Paris France; 2 Université Paris Cité Laboratoire de Psychopathologie et Processus de Santé Boulogne-Billancourt France; 3 Centre de l’insomnie PsySomnie Toulouse France; 4 Centre National de la Recherche Scientifique (CNRS) Institut du Cerveau-Paris Brain Institute, Institut National de la Santé et de la Recherche Médicale U112, iCrin Inserm Paris France; 5 Department of Psychiatry Saint Antoine Hospital, DMU Neurosciences, Assistance Publique-Hôpitaux de Paris (AP-HP) Sorbonne Université Paris France; 6 Di&Care Loos France; 7 Département de psychiatrie et d'addictologie, Assistance Publique-Hôpitaux de Paris (AP-HP) DMU Neurosciences Hôpital Bichat-Claude-Bernard Paris France; 8 NeuroDiderot, F-75019 Université Paris Cité Inserm Paris France

**Keywords:** digital program, insomnia, integrative therapies, CBT-i, nonpharmacological intervention

## Abstract

**Background:**

Chronic insomnia, or insomnia disorder, is a major health issue with a prevalence of up to 15%. The recommended first-line treatment is cognitive behavioral therapy for insomnia (CBT-i), which, unfortunately, remains insufficiently accessible. Digitalization has the potential to reduce health care access inequalities by offering more flexible and accessible care options. Digital CBT-i (dCBT-i) has been shown to be as effective as in-person CBT-i, highlighting its potential for broader implementation.

**Objective:**

This study aimed to develop an evidence-based dCBT-i program grounded in theoretical and clinical knowledge, designed for efficient integration into health care systems, and to establish it as the first prescribed digital treatment in France.

**Methods:**

The program was constructed based on validated CBT-i theory and practice, incorporating the latest scientific data on CBT for insomnia. It was designed as a robust multicomponent therapy, integrating an initial standardized assessment and daily intelligent adaptation to enable digital phenotyping and provide personalized treatment.

**Results:**

We developed an innovative digital solution that combines scientific rigor with practical application. The program includes a standardized initial evaluation and dynamic personalization through intelligent algorithms. These features allow for the adaptation of therapy based on patient progress and needs, ensuring individualized care.

**Conclusions:**

The development of this dCBT-i program represents a significant milestone in digital health care, offering a scalable solution to the accessibility challenges of traditional CBT-i. Future steps involve conducting clinical studies to further evaluate its effectiveness and optimize its implementation within health care systems.

## Introduction

Insomnia is a major health complaint, with a prevalence up to 50% in primary care patients undergoing current treatment for either independent insomnia or insomnia comorbid with another disease, posing a risk of worsening comorbid medical conditions without addressing insomnia [1]. Chronic insomnia, or insomnia disorder, has an estimated prevalence of approximately 9% to 15% based on criteria from the Diagnostic and Statistical Manual of Mental Disorders (Fourth Edition; DSM-IV) and the International Classification of Sleep Disorders [2]. Moreover, insomnia affects 85% of patients with a mood disorder during depressive phases, and more than one-half of the patients will retain insomnia symptoms after remission [3,4]. Insomnia disorder is defined by sleep dissatisfaction for at least 3 months and 3 nights per week despite adequate opportunity for sleep, with difficulty initiating sleep, maintaining sleep continuity, early morning awakening, or poor sleep quality and with daytime dysfunction and significant impairment in areas of functioning [5,6].

The latest European Insomnia Guideline suggests that cognitive behavioral therapy for insomnia (CBT-i) is the reference and first-line treatment for insomnia disorder, with sustained improvements over time [7], and multicomponent CBT-i is strongly recommended [8]. Another important aspect of recent research involves distinguishing between response and remission rates. Response is typically defined as a significant reduction in Insomnia Severity Index (ISI) scores, while remission is generally defined as achieving a final ISI score ≤7. Studies have shown that 70% to 80% of patients respond positively to CBT-I, with about 50% of patients showing a clinically meaningful reduction of symptoms, while remission was attained by approximately 40% of patients receiving CBT-i and outcomes were often improved at the 6-month follow-up for both response and remission [7,9-12]. In addition, patients who do not respond well to CBT-i often have insomnia associated with a mood disorder and with insomnia complaints often masking disruptions in the biological clock or a state of “hyperarousal” [1,7,13]. However, CBT-i has been shown to be effective in treating insomnia comorbid with a mood disorder [14] and to also have an effect on psychiatric symptoms comorbid with insomnia disorder [15,16], and mindfulness techniques (often integrated into the emotional regulation component of CBT-i) were found to reduce hyperarousal [17-20].

CBT-i has largely been proven effective but is not sufficiently accessible as they are too few CBT-i therapists available and the treatment can be expensive [7,21-24]. Digital CBT-i (dCBT-i) could make treatment more accessible. Digitalization of CBT-i was first proposed in the 1990s, and the first proposal to introduce dCBT-i into the health care system as a preliminary step before in-person orientation was made in 2009 [25]. There are now numerous programs with proven efficacy and technological advances that allow for adaptation and personalization of content, while some obstacles still reside, including patient adherence and the perception of individualization [21-24,26-28]. Some programs are fully automated, while others are guided (mixing human and digital interaction) or only used as a tool to support in-person care [29]. To make CBT-i more accessible, fully automated treatments seem to be more relevant, but they are considered less engaging and associated with poorer adherence. The latest technological advances, encouragement for therapeutic adherence, and digital phenotyping to create a sense of treatment personalization should allow innovative, fully automated programs to be as engaging as in-person care [21-24,26-28,30]. dCBT-i has already been proven effective at reducing insomnia severity, with many studies reporting significant reductions in ISI [31] scores after treatment compared with control groups. For instance, a study reported an average difference in ISI score of 7.6 points, while a meta-analysis of 33 randomized controlled trials (RCTs) found a mean difference of 5.0 points between treatment and control groups [32,33]. dCBT-i was also found to have a significant effect on quality of life and cognitive functions, and efficacy was also demonstrated for mild insomnia or insomnia comorbid to a mood disorder [34-38]. dCBT-i was also found to have a reducing and protective effect on comorbid psychiatric symptoms [39-44] and a preventive effect on sleep disturbances after a stressor such as during the COVID-19 pandemic [45].

Digital interventions could significantly reduce access inequalities by introducing less stigma, being more flexible, and being less expensive for the patient than in-person therapy [23,34,46]. dCBT-i could then be a perfect solution to the challenge of access, as it was proven to have the same efficacy as in-person CBT-i. As highlighted in a recent meta-analysis, dCBT-i may be a viable alternative when in-person care is not available [47]. However, some issues still remain. First, many interventions exist, but less than 15% of the available “care apps” are based on strong theoretical models [23,48]. Second, adherence is an important challenge with this type of care, as less than 30% of users participate in digital programs in their entirety [30,49,50]. Finally, evidence-based, valid programs are not always made available to the public, as they must be implemented in the health care system [51].

In this context, our goal was to describe the 2-year development of an evidence-based digital program that could retain CBT-i efficacy while being accessible, engaging, and ready to be implemented in the French health care system, as already accomplished in other countries [52,53]. We further sought to develop an innovative digital solution grounded in the latest scientific data on CBT-i, with a robust multicomponent therapy approach. This solution integrates an initial standardized assessment and daily intelligent adaptation, where digital phenotyping and algorithms adjust the treatment based on daily monitoring of the patients’ behaviors, optimizing the treatment in real time and providing personalized solutions. This cutting-edge treatment is grounded in the latest scientific data and uses technological tools to offer individualized and evolving treatment based on a robust standardized assessment of the patients’ sleep complaints. Furthermore, algorithmic possibilities and artificial intelligence (AI) should allow the treatment to evolve continuously and adapt to the changes in the patients’ daily lives, making the treatment even more patient-centered and tailored to its specific needs than most traditional CBT-i protocols.

## Methods

### Program Overview

We developed a digital program tailored for French patients diagnosed with insomnia disorder by their medical practitioner, integrating data from the evidence-based CBT-i literature, user research, and clinical expertise. The development process followed the latest guidelines provided by various authors specializing in digital intervention issues and the treatment of insomnia disorder [7,8]. To make the app useful and safe, we followed the ASPECTS (Actionable, Secure, Professional, Evidence-based, Customizable, Transparent) guidelines and the various steps used in evaluating an app [54].

Our method should allow prescribers to integrate into their care useful data and information about their patient from our intervention such as a sleep diary and daily patient habits.

The French guidelines for safety and privacy were followed, and the data will be secured and protected according to those guidelines.

Various experts (eg, legal advisors, scientific and medical professionals, sleep practitioners, psychologist specializing in CBT-i, digital and user experience design specialists) whose credibility and capability were assessed were included in the process.

We conducted a deep literature review, and the entire program was based on CBT-i as described in the literature. In addition, dCBT-i is already evidence-based [7].

The program is flexible and adaptable to its users, and the app should be easy enough to be engaging, which will be determined though user research and planned studies. The program provides transparency to users about how it works and collects data. Electronic sessions (e-sessions) mimic real-life CBT-i consultations where the patient is introduced to fundamental tools and information, and every recommendation is explained well to the patient in order to develop good treatment adherence.

The program was built according to known psychological models of change in order to “reflect the psychological chronology of the change process” [55].

Finally, the program is personalized for each patient using numerous tests and algorithms, which define a tailored treatment pathway for the patient's insomnia.

**Figure 1:**
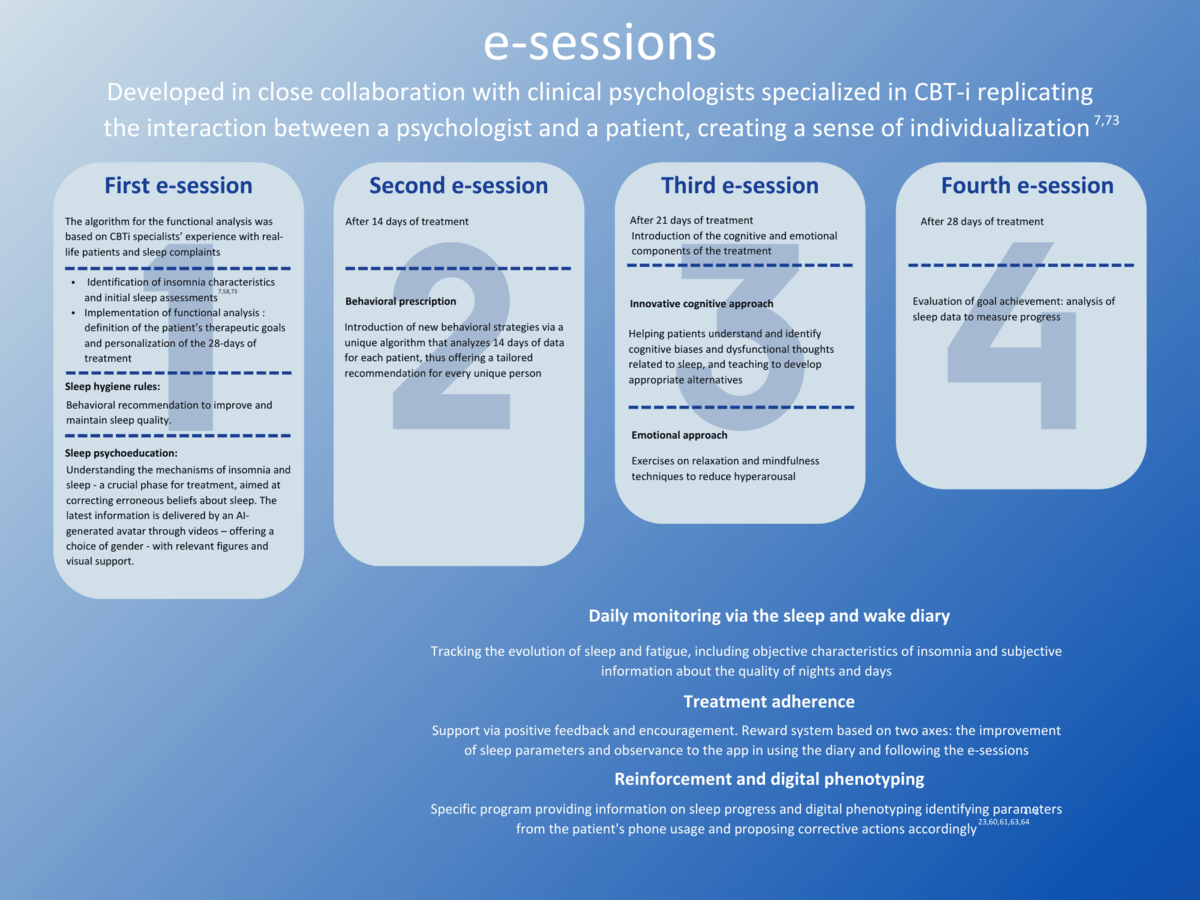
Key components of the digital insomnia treatment program. AI: artificial intelligence; CBT-i: cognitive behavioral therapy for insomnia; e-session: electronic session.

The digital medical device Noxxea will undergo clinical validation to demonstrate its effectiveness and safety of use with patients with insomnia. The method incorporates traditional pillars of insomnia management through several e-sessions designed to replicate the interaction between a psychologist and a patient (Figure 1).

The first e-session involves identification of the patient’s insomnia characteristics and initial sleep assessments, based on the requirements of an in-person clinical evaluation and traditional theoretical models of insomnia [7,56]. Implementation of functional analysis involves personalization of a 28-day treatment based on this analysis and defining the patient’s therapeutic goals. The algorithm was based on CBT-i specialists’ experience with real-life patients and sleep complaints.

Daily monitoring via the sleep diary includes use of the sleep and wake diary. The diary tracks the evolution of sleep and fatigue, including objective characteristics of insomnia and subjective information about the quality of nights and days.

Sleep hygiene rules include behavioral recommendations to improve and maintain sleep quality.

Sleep psychoeducation helps to understand the mechanisms of insomnia and sleep. This is a crucial phase for treatment, aimed at correcting erroneous beliefs about sleep. The latest information is delivered by an AI-generated avatar in videos with relevant figures and visual support.

The second e-session (after 14 days) is the mid-treatment assessment, which evaluates the first 14 days of treatment. The behavioral prescription introduces new behavioral strategies via a unique algorithm that analyzes 14 days of data for each patient, thus offering a tailored recommendation for every unique person.

The third e-session (after 21 days) involves cognitive and emotional restructuring. Although this approach is quite rare in digital formats today, it remains essential for CBT-i. Thought management was finally made possible in dCBT-i through a specific module based on Beck’s cognitive restructuring techniques [57]. It enables patients to understand cognitive biases and dysfunctional thoughts, identify automatic thoughts related to sleep, and learn to respond to them with appropriate alternatives. Another module offers exercises on relaxation and mindfulness techniques to reduce hyperarousal.

The final assessment (after 28 days) evaluates achieved goals, including an analysis of sleep data to measure progress.

To ensure adherence to treatment, a reward system was developed based on 2 axes: improvement of sleep parameters and adherence to entering the agenda and e-sessions in the app.

Regarding reinforcement and digital phenotyping, throughout the treatment, the patient receives reinforcement through a specific program providing information on sleep progress, thus facilitating engagement. An essential aspect of the app is the use of digital phenotyping, which identifies parameters from the patient’s phone use and proposes corrective actions accordingly.

The inclusion of digital phenotyping is a key element of the method that allows for further adaptation and personalization of treatment and makes it as close to in-person care as possible. In 1982, evolutionary biologist Richard Dawkins highlighted the arbitrary nature of many phenotypic delineations and introduced the concept of the “extended phenotype” [58]. This concept suggests that phenotypes should include all effects a gene has on its environment, not just biological processes like protein biosynthesis or tissue growth. Both animals and humans modify their surroundings, and these modifications, along with associated behaviors, are expressions of their genomes, forming part of their extended phenotype. Dawkins used the dam-building behavior of beavers as an example of an extended phenotype. With the increasing integration of personal technology into human lives, a novel concept called digital phenotyping was introduced in 2015 by Jain et al [59] and further developed by Torous et al [23,60,61] in psychiatry. Digital phenotyping involves real-time collection of data and markers of human behavior to identify the “digital signature of a pathology.” It assumes behaviors can be quantified through data from connected tools like smartphones, digital sensors, and wearable devices to derive an “e-semiology” [62]. Two types of data are considered. “Passive” data are automatically collected in real time without user input, using tools like accelerometers and GPS to detect psychomotor retardation or motor agitation. Deviations in GPS coordinates from typical travel patterns, such as reduced activity, increased time spent at home, or chaotic journeys to unusual locations, may indicate pathological behaviors. Mobile phone–based sensing software measures heart rate, heart rate variability, galvanic skin conductance, and blood pressure, which are regarded as potential biomarkers of certain psychiatric conditions. Supplementing this with “active” data is essential, as metrics like screen time, number of calls, and usage hours provide valuable indicators of psychosocial functioning [23,60,61]. Additionally, analyzing language (verbal and paraverbal) and emotional arousal offers insights into the valence of messages during social interactions, indicating the user’s mood and mental state. Digital phenotyping can also help minimize cognitive biases, such as confirmation or self-serving biases, by collecting objective information through passive data, for instance. This approach is highly promising for mental disorders and allows for optimal therapeutic personalization, almost in real time, and enables the enhancement of patient empowerment [63,64].

### Noxxea Development

Several steps described here were carried out to create this innovative solution based on the latest scientific data.

Treatment development was first discussed with experts in the field in order to take patient needs into account from the beginning. As a result, there was a dynamic interplay between scientific research and product development.

An acceptability study validated certain ergonomic aspects of the app and the usefulness of generative AI for sleep solutions (M Villechevrolle, unpublished data, May 2025). A study involving 582 users was conducted to gather their concerns and feedback. It used the script method, allowing for both qualitative and quantitative analysis of user reactions. The findings were then integrated into the ongoing development of the app to enhance user trust and ensure data privacy.

The most important aspect of the development was to remain as close to in-person treatment as possible.

Translating CBT-i into a digital format requires maintaining the relational aspect of therapy. Indeed, in psychotherapy, a form of therapeutic alliance is essential, and it is not yet fully known how that translates to a digital program [30]. Flexibility and personalization of treatment seem fundamental—for instance, dCBT-i with a therapist or direct context with a practitioner or therapist-guided dCBT-i had greater effects on sleep parameters and treatment adherence than dCBT-i without a therapist [38,65-67]. Programs are effective even without support, but better results with digital programs are found when the users have a sense of support and individualized care [22,68-71]. This may also help explain why a recent network meta-analysis of various CBT-i delivery methods found that both guided and unguided dCBT-i achieved medium effect sizes, while smartphone-based CBT-i did not show significant effects compared with the control group [7,47].

Therefore, a significant effort was made to develop a form of communication within the app that was as close to an in-person consultation as possible. The participant can choose the avatar’s gender, and support is offered through positive feedback and encouragement via the app. A reward system was developed to engage the patient and help him or her identify changes and improvements. Every item of text and dialogue was written by clinical psychologists specializing in CBT-i in the same way a therapist would talk to his or her patient, and the e-sessions were developed to recreate a sense of individualization and the 4 to 8 validated sessions of CBT-i [7,72].

Nonetheless, we prioritized chronobiological regulation with emotional and cognitive aspects to stay true to the description and validation of CBT-i in the literature, which does not require the same relational demands in terms of therapeutic alliance as in-person psychotherapy (which typically requires a higher number of sessions and is often more suitable for resistant insomnia). The program was thus developed according to the components described in sleep recommendations and many studies using this form of treatment for sleep disorders [7,72,73].

### Psychoeducation and Sleep Hygiene

The program must address the sleep hygiene “rules.” Therefore, explanations about recommendations, habits, sleep functioning (roles and functions as well as circadian patterns), and changes in sleep patterns across the lifespan are given and integrated into the treatment.

### Behavioral Treatment

A sleep calendar is introduced at the beginning of the treatment to collect at least 10 nights of sleep data in the first 2 weeks of treatment, allowing the calculation of individualized sleep parameters and habits. Sleep restriction and stimulus control are the main behavioral strategies used in the program. These components were developed in close collaboration with the scientific committee and translated into a digital format within the app in accordance with established CBT-i guidelines. Sleep restriction is thus personalized for each patient according to an individualized sleep calendar, and stimulus control is personalized using patient-reported data, the chronotype, and routines assessed at the start of the program. Personalized video content targeting chronobiological rhythms and behavioral habits further supports the tailoring of treatment to each patient’s specific needs.

### Relaxation Component

Many different relaxation strategies as well as mindfulness meditations are introduced within treatment as ways to reduce somatic tension, arousal, or intrusive thoughts. Patients are encouraged to explore them during their treatment.

### Cognitive Component

Cognitive strategies are introduced as ways to identify and challenge misconceptions and faulty beliefs about sleep and state during the day. Patients are introduced to the concepts of cognitive control at bedtime and cognitive restructuring to learn to challenge thoughts. They are encouraged to explore these strategies within the framework of behavioral change.

Developing this program was challenging due to the need to incorporate sleep medicine recommendations using a digital intervention while designing it to closely resemble an individualized care format. The presented treatment not only offers sleep education, which is predominantly present in therapeutic education programs in institutions or digital platforms, but also integrates an individualized functional analysis adapted for digital use. It guides the patient toward a form of cognitive therapy similar to that provided in CBT-i treatment.

## Results

The Noxxea program is now fully developed, and several steps are planned for its clinical evaluation, including an RCT scheduled for 2025 that will evaluate the program against a waiting list control group.

### Digital Phenotyping Research

Collaboration with the mathematics department at the Pasteur Institute and the Institut National de la Santé et de la Recherche Médicale (INSERM) research laboratory (Decision and Bayesian Computation, Computational Biology & Neuroscience Department, Centre National de la Recherche Scientifique [CNRS] Unité Mixte de Recherche [UMR] 3571 – Jean Baptiste MASSON) will allow analysis of data to enhance the treatment’s algorithms and improve efficacy at predicting and preventing insomnia relapse. This work will mainly focus on digital phenotyping: Approximately 50 data points will be collected and analyzed to aid with the detection, prediction, and prevention of chronic insomnia relapses. The digital phenotyping initiative is central to the innovative dCBT-i and has the following 3 main objectives: validate the initial selection of the 4 key tracking data points chosen for the first version of the treatment; analyze and evaluate the 70 passive and active data points collected to determine interaction models that enable detection of symptoms, prediction, and prevention of insomnia relapse, as well as the isolation of additional data that could enhance the efficacy of a new version of the treatment and improve adherence; and test a second version of the treatment by validating the selection of new tracking data points.

### Effectiveness of Real-World Implementation and RCT

We will evaluate the efficacy and compare it with that of in-person CBT-i in the same population. The WHAT (work, help, accessible, trusted) criteria can be used to evaluate a digital intervention, and we will need to evaluate the following [51,74]:

- Work: To evaluate efficacy, we plan to conduct an RCT.
- Help: A clinically significant effect in the target population would help conclude that it can be a useful addition to in-person CBT-i.
- Accessible: Technical quality will be continuously assessed (by professionals and users).
- Trusted: Risk management and adherence to national and international standards and guidelines will be assessed and continuously updated to the latest requirements.

## Discussion

This dCBT-i treatment is built according to European recommendations and guidelines and follows closely what is done in person. The goal is not to replace in-person care but to offer an alternative when in-person care is not available given that not all individuals with insomnia disorder can receive the recommended treatment in France. Ideally, it will be covered by the national health insurance system as it is a first-line medical treatment for a major health issue.

The digital form of insomnia disorder treatment incorporates validated and recommended behavioral, cognitive, and emotional techniques for insomnia, closely mirroring the CBT-i treatment offered traditionally while being even more tailored to patients’ individual needs. It is meant to be a medical treatment prescribed by a health care professional following an evaluation and a precise diagnosis, addressing the dual challenge of considering therapeutic adherence and support in its development while individualizing the behavioral and cognitive components of the treatment for greater effectiveness. The innovative program presented here followed those demands, as well as safety and security guidelines for digital health interventions while being grounded in the latest technological advances and scientific data on CBT for insomnia. It would offer access to an evidence-based multicomponent treatment and is intended to be integrated into the health care system as such. In line with expert recommendations, the Noxxea solution could ideally be part of a stepped care model that adapts to the severity of the condition and the patient’s needs, with dCBT-i proposed as a first step delivered through multiple pathways, such as being prescribed by a doctor or accessed directly by the patient [51].

## Abbreviations

ASPECTS: Actionable, Secure, Professional, Evidence-based, Customizable, Transparent
CBT-i: cognitive behavioral therapy for insomnia
CNRS: Centre National de la Recherche Scientifique
dCBT-i: digital cognitive behavioral therapy for insomnia
DSM-IV: Diagnostic and Statistical Manual of Mental Disorders (Fourth Edition)
INSERM: Institut National de la Santé et de la Recherche Médicale
ISI: Insomnia Severity Index
RCT: randomized controlled trial
UMR: Unité Mixte de Recherche
WHAT: work, help, accessible, trusted
